# Optical Tecnology Developments in Biomedicine: History, Current and Future

**Published:** 2011-10-17

**Authors:** Shoko Nioka, Yu Chen

**Affiliations:** 1 Department of Biochemistry and Biophysics, Perelman School of Medicine, University of Pennsylvania, Philadelphia, PA 19104 USA - shoko@nioka.net; 2 Fischell Department of Bioengineering, University of Maryland, College Park, MD 20742 USA - yuchen@umd.edu

## Abstract

Biomedical optics is a rapidly emerging field for medical imaging and diagnostics. This paper reviews several biomedical optical technologies that have been developed and translated for either clinical or pre-clinical applications. Specifically, we focus on the following technologies: 1) near-infrared spectroscopy and tomography, 2) optical coherence tomography, 3) fluorescence spectroscopy and imaging, and 4) optical molecular imaging. There representative biomedical applications are also discussed here.

## 1. Introduction

Optical technologies are currently emerging as promising tools for medical imaging and diagnostics. Optics has several advantages, including non-ionizing radiation, low-cost, portable, and high molecular and biochemical specificity. These advantages enable functional imaging using light and open up new opportunities for light-based applications in clinical medicine.

This paper reviews several optical technologies that have been developed and translated for either clinical or pre-clinical applications. Specifically, we focus on the following technologies: 1) near-infrared spectroscopy and tomography, 2) optical coherence tomography, 3) fluorescence spectroscopy and imaging, and 4) optical molecular imaging. There representative biomedical applications are also discussed here.

## 2. Review of Optical Technology

### 2.1 Near-infrared (NIR) Spectroscopy and Tomography

#### Historical Perspective and Technology Development

Biological molecules have unique absorption spectra against a range of light wavelengths, thus can be detected with accurate concentration by spectroscopy. The spectroscopy system was pioneered in the Cambridge University [1, 2]. However, biological sample is usually opaque and therefore the light absorption spectra would be disrupted due to scattering. In 1950, Britton Chance invented “double beam spectrometer” using two wavelengths in the visible region with a small spectral interval to eliminate the effect of scattering [3]. This double beam concept was adapted to the optical spectroscopy used for biological systems even up to date for medical use.

Pulse oximeter may be the first use of optics for human in vivo, which utilizes near-infrared light to monitor arterial hemoglobin oxygen saturation. It was first made by Takuo Aoyagi in a Japanese company, Nihon Kouden in 1972 [4]. Because arterial pulse induces changes in arterial blood volume between systolic and diastolic heart contraction, light intensity difference between these two conditions is only caused by arterial blood. Thus arterial blood oxygen saturation can be quantified with a simple linear equation [5], ignoring scattering effects of tissue. This concept is similar to the “double beam spectroscopy” dated back to 1940s, when Glenn Milliken tried to observe differences of light transmitting intensity through human tissues using green and red color filters to measure oxygenation in human tissue [6].

**Figure 1:**
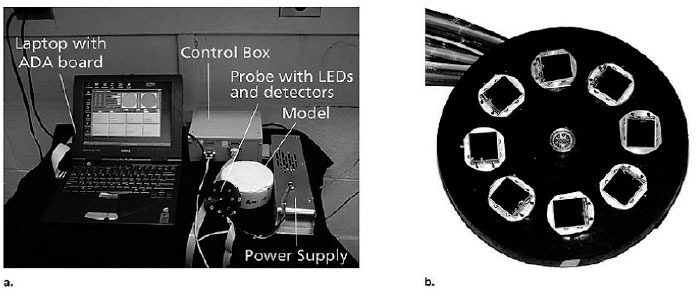
A photograph of the whole apparatus (a) illustrates the handheld puck or probe, the coupling to the circuit box which contains the drivers for the LED, the amplifiers for the detectors, the digitally controlled gain adjustment amplifier, the electronic switch which decodes the light pulses and stores the information in a memory capacitor, the second set of switches which sample the memory capacitor at a rate compatible with the computer analog-to-digital converter (ADC), and (b) Handheld puck. From Ref. [8], with permission.

The first demonstration of NIR light on human tissue in vivo was reported by Franz Jobsis in 1977 [7]. Jobsis demonstrated that NIR light can carry information of not only hemoglobin but mitochondrial chromophore, cytochrome a,a3 in the neonatal brain measured non-invasively. Since then, many papers were published along the line of proving tissue oxygenation and mitochondrial redox states by means of those hemoglobin and cytochrome a,a3 signals in the NIR region in many animal models and human tissues. Many researchers use continuous-wave (CW) technology as the system is simple, low-cost, and robust. Figure 1 shows a representative CW near-infrared (NIR) imaging system [8] with three-wavelength light emitting diode (LED) at 760 nm, 805 nm, and 850 nm, and 8 silicon photodiode detectors. Many companies such as Somanetics have commercialized this technology to measure tissue hemoglobin saturation. Since only intensity attenuation is measured with CW system, it is difficult to separate scattering coefficient from absorption coefficient in the tissue.

Time-Resolved Spectroscopy (TRS) technology gave a solution for this problem of absolute quantification of chemical concentrations in the turbid media such as the in vivo human investigation in 1988–1989 by B. Chance [9, 10]. TRS machines are commercially available then in 1993 by Hamamatsu, and it has been made for many applications [11, 12]. Alternatively frequency-domain (FD) NIR spectroscopy (NIRS) can be also used for quantization [13–15]. FD technology is available commercially by ISS, Inc. These two technologies have been used for obtaining more accurate information from the turbid tissue, namely absorption and reduced scattering coefficients.

NIRS can be extended to imaging mode by using multiple source-detector channels. One way to form an image is using back-projection and interpolation algorithms. This approach, sometimes referred to as diffuse optical topography, can provide a quick and good estimate of 2D spatial distribution of the optical properties of interested. The drawback of this relatively simpler approach is that the tissue optical properties are not reconstructed with good accuracy, and the spatial resolution is lower [16]. Another approach is to perform 3D tomographic reconstruction, therefore, is referred to as diffuse optical tomography (DOT). In principle, DOT is similar to other tomographic schemes such as X-ray computerized tomography, and involves image reconstruction by solving the inverse problem [17]. DOT can accurately reconstructed the spatially-resolved changes in optical properties in tissue.

#### Clinical Applications of NIR

NIR diffuse optical spectroscopy and imaging techniques have been applied to numerous clinical applications. Here we focus on three main application areas, namely: 1) breast cancer detection and characterization; 2) functional brain imaging; and 3) imaging the skeletal muscles.

##### Breast Imaging

Breast cancer is the most commonly diagnosed cancer among women in the United States and worldwide. Early detection through mammography and clinical breast exams is essential for effective breast cancer screening. For women between the ages of 50–69, regular mammograms can reduce the chance of death from breast cancer by approximately 30% [18]. X-ray mammography may miss up to 25% of breast tumors in women in their 40s, and about 10% of women over age 50. Other imaging techniques, such as magnetic resonance imaging (MRI) and ultrasound (US), have been developed for breast cancer detection and staging without using X-rays [19, 20]. In general, mammography, MRI and US provide mostly anatomic information, rather than quantitative tissue function and composition [21]. Positron Emission Tomography (PET) could provide the metabolic information, but requires the injection of exogenous radionuclides [22].

Compared with those modalities, NIR diffuse optical imaging has its own merits of non-ionizing, economic and biochemical specificity. The use of light in breast cancer detection dates back to the 1920s [23]. In the past two decades, with the development of advanced light sources and detector, as well as modeling of light propagation in tissue, the application of diffuse optical imaging for breast imaging (often referred to as “optical mammography”) have been developing rapidly. The development of tumor is generally associated with increased vascularization (also called “angiogenesis”) [24, 25] and lower oxygen partial pressure (pO_2_) [26, 27]. NIR light is sensitive to intrinsic chromophores such as oxygenated and deoxygenated hemoglobin (HbO_2_ and Hb), water (H_2_O) and lipids [28, 29]. Therefore, NIR diffuse optical spectroscopy and imaging can provide sensitive and specific physiological information for breast cancer diagnosis [30, 31]. Nioka et al introduced endogenous contrast NIR imaging of the human breast in 1994 [32]. Blood volume and oxygen saturation are two important parameters. Studies indicate that there are two to four folds of contrast between normal and tumor regions for blood volume, and oxygen saturation in the tumor is generally lower than normal [21, 29, 31]. There exist variations in normal breast tissue optical properties. For example, Durduran et al reported the averaged blood volume and oxygen saturation on healthy female breast tissues are 34 ± 9 μM and 68 ± 8 %, respectively [33]. These baseline values are important to assess the potential contrasts available for diffuse optical imaging to discriminate healthy and diseased tissues. The scattering properties of tissue also contain important information for lesion diagnosis. The scattering coefficients are related to the tissue structure properties and the concentration or size of organelles [34].

The clinical niche for NIR diffuse optical spectroscopy and imaging in breast cancer are tumor detection in pre-menopausal women and monitoring neoadjuvant chemotherapy [35–37]. CW systems are relatively inexpensive and compact. It can be interfaced with a handheld probe to image the breast. Using the handheld puck shown in Figure 1, Chance et al reported a 6 year, two-site study on 116 patients of whom 44 patients had confirmed malignancy by biopsy and histopathology [8]. The absorbance increments of the cancerous regions are referred to the mirror image location on the contra-lateral breast. This technique was able to distinguish cancer from non-cancer breasts with 96% sensitivity and 93% specificity. In another pilot clinical trial of 48 patients, Xu et al used a portable handheld NIR imaging device, “P-Scan”, a pre-commercial prototype of ViOptix Inc. (Fremont, CA, USA), to image suspicious breast lesions identified on diagnostic clinical ultrasound (US) [38]. An external mechanical compression was applied to breast tissue to dynamically record the oxygen saturation and hemoglobin concentration.

**Figure 2:**
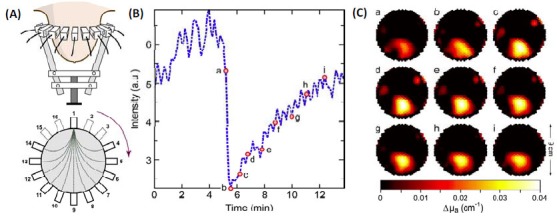
(A) CW imager configuration. The sources are sequentially shining upon the breast and so the configuration is equivalent to a fan-beam configuration. (B) The dashed curve represents the intensity drop associated to the ICG-uptake for source 6 and detector 3 (not capturing mass area). (C) Differential absorption reconstruction for the time selected in (B). From Ref. [40], with permission.

Indocyanine Green (ICG) is an organic dye that has been approved by US Food and Drug Administration (FDA) for clinical use. ICG can be used to enhance the tumor-to-normal contrast to aid in the detection of lesions in the breast, as first demonstrated by Nioka et al [39]. Figure 2 shows an example of CW DOT system for imaging the uptake of ICG by breast tumors [40]. This instrument employs a circular configuration with 16 sources and 16 detectors (Figure 2A) to collect light in parallel on the surface of the tumor-bearing breast. ICG was injected by bolus, and the absorption changes induced by ICG were recorded dynamically (Figure 2B). DOT successfully localized the tumor regions, which was in good agreement with a priori information (Figure 2C). A two-compartment model composed of plasma and extracellular-extravascular space was used to analyze the pharmacokinetics of ICG. Moreover, different dynamical features were observed for different pathologies. The malignant cases exhibited slower rate constants (uptake and outflow) compared to healthy tissue. Further studies enabled direct forming of the pharmacokinetics-rate image by DOT, and found statistically different rates from the tumor region compared to those outside the tumor region [41]. These results demonstrated that in vivo pharmacokinetics of ICG in breast tumors could be a useful diagnostic tool for differentiation of benign and malignant pathologies. Schmitz et al developed a more sophisticate CW system for bi-lateral breast imaging, DYnamic Near-Infrared Optical Tomography (DYNOT) system (NIRx Medical Technologies) [42]. This system enables simultaneous imaging of hemodynamics within both breasts.

Frequency-domain measurement can quantify the concentration of chromophores. Culver et al developed a hybrid CW/frequency-domain breast imaging system which combines the benefits of high-speed and low-cost of CW techniques with more accurate quantitative nature of frequency-domain techniques [43]. Using this system, Choe et al [44] found that malignant cancers (n=41) showed significantly higher total hemoglobin, oxy-hemoglobin concentration, and scattering compared to normal tissue. Benign tumors (n=10) did not exhibit statistical significance in the tumor-to-normal ratios of any parameter. These results demonstrate that benign and malignant lesions can be distinguished by quantitative three-dimensional DOT. Such a system also has been applied to monitor neoadjuvant chemotherapy [45]. DOT revealed tumor shrinkage and decrease in relative total hemoglobin concentration during the course of chemotherapy, therefore demonstrated the potential for monitoring physiological parameters of breast lesions during chemotherapy. Tromberg’s group at UC Irvine also developed a handheld NIR diffuse optical spectroscopic imaging (DOSI) system for breast cancer detection and monitoring neoadjucant chemotherapy [37].

Time-domain DOT systems have been also developed for breast imaging. Ntziachristos et al developed a time-domain imaging system using time-correlated single photon counting (TCSPC) technique, and demonstrated concurrent MRI and DOT imaging of breast after contrast enhancement using ICG [11]. Other prototype instruments have been developed by groups at Politecnico di Milano, Italy [46–48] and Physikalisch-Technische-Bundesanstalt of Berlin, Germany [12, 49–51], as part of Optimamm, a consortium funded by the European Union, and have acquired data from more than 300 clinical cases. They reported successful identification of 80%–85% mammographically identified lesions. A prototype time-domain DOT breast imaging system has been developed by Advanced Research Technologies (ART, Canada) [52]. Initial results suggested that optical imaging can discriminate benign and malignant tumors, therefore, held great clinical promise for breast cancer imaging.

One of the recent trends in NIR DOT is to combine with other imaging modalities such as X-ray CT, MRI or US, which can provide high spatial resolution map of tissue structures. Those maps can be used as a priori information to improve the reconstruction of DOT images [53, 54]. Representative multi-modality breast imaging systems include the combined DOT and X-ray mammography system developed at the Massachusetts General Hospital [55, 56], the combined DOT and MRI multi-modal imaging system developed at Dartmouth College [53, 57], and the combined ultrasound and frequency-domain diffuse optical imaging/tomography systems [58–61].

##### Brain Imaging

Since Seiji Ogawa’s discovery that deoxyhemoglobin signal changes in NMR can detect brain cognition in early 1990s [62], researchers are interested in using NIR light to detection brain function [63–65]. NIRS can quantify the concentration of both Hb and HbO_2_, thereby revealing the blood volume and oxygenation saturation changes associated with brain functions.

NIR diffuse optical imaging (DOI) has found widespread applications in clinical settings [66, 67]. One major research area is to understand how the brain functions. DOI offers unique capability to non-invasively monitor the functional activations in vivo without disturbing the organ. Various applications such as visual responses [68, 69], somatosensory responses [70], auditory responses [71], language stimuli [72, 73], and problem solving [74] have been explored. Another important area for DOI brain imaging is to diagnose and monitor the diseases such as stroke [75, 76], epilepsy [77], brain injury [78], and post-traumatic stress disorder [79]. Optical techniques are well-suited for early detection of hemorrhage [80], and discrimination between ischemic and hemorrhagic stroke leading to a better management of the patient treatment [81].

Commercial CW brain imaging systems have been developed by Hitachi Medical Corporation (Tokyo, Japan) [82, 83]. This optical topography system (ETG-100) uses 8 laser diodes at 780 nm and another 8 at 830 nm. 8 avalanche photodiodes (APDs) are used to detect the signals. Multiple channels are encoded by different frequencies from 1 to 6.5 kHz. The Hitachi system has been applied to investigate healthy brain functions such as language development [72], the emotional response to music [84], cognitive functions [73, 85], and brain development in newborn infants [86, 87], as well as pathological conditions such as epilepsy [77], post-traumatic stress disorder [79], and cognitive function study in patients with motor neuron disease [88]. The clinical applications of the Hitachi system have been quite successful despite using a simple CW system and relatively simple image reconstruction method. Other companies such as Shimadzu Corporation (Japan) also developed optical topography system from brain imaging [89].

Franceschini et al reported the development of the CW imaging system (CW4 and CW5) at the Massachusetts General Hospital [90, 91]. The newer system (CW5) employs 32 sources and 32 detectors to cover most of the areas in the adult head, which enables simultaneous collection of optical signals from prefrontal, sensorimotor, and visual cortices in both hemispheres. Using this system, they investigated the spatiotemporal patterns of physiological signals during rest. This information will help to understand the physiological signals and develop signal processing algorithms to distinguish them from the functional activation signals [91]. White et al applied a high-performance, high-density CW DOT system [69] to map resting-state networks in the human brain [92], which enables studies of the functional connectivity of different cortical regions. These studies demonstrated that high-density DOT has become a practical and powerful tool for mapping function in the human cortex.

ISS Inc. has developed a commercial frequency-domain brain imaging system (Imagent™). Frequency-domain DOI has been shown to measure the hemodynamic (slow) signals [93, 94] and neuronal (fast) signals [95, 96]. Especially, the fast signals are thought to be associated with the neuronal scattering changes, which will induce phase delay in the modulated diffuse photons. Therefore frequency-domain imaging system is required to measure the phase delay which indicates the event-related optical signals (EROS). Time-domain imaging systems have been actively developed for brain imaging, especially for tomographic imaging of whole brain. Hintz et al reported the early development of time-domain optical tomographic system for neonatal brain imaging by reconstructing measurements of mean photon flight time [97]. More recently, the group at University College London (UCL) has developed a 32-channel time-resolved optical imaging system [98] for 3D neonatal brain imaging. This system has been successfully used to image the brain of a premature infant with a cerebral hemorrhage [99] and monitor the blood volume and oxygenation changes in the newborn infant brain during ventilation [100].

##### Muscle Imaging

Non-invasive monitoring of muscle tissues using light can be dated back to the 1930s by Millikan [101]. Since then, optical imaging of muscles has received steadily increased interests. Optical methods can probe hemoglobin, myoglobin, blood flow, and metabolism, therefore provide an ideal means for monitoring muscle functions under different physiological or pathological conditions [102–106].

**Figure 3:**
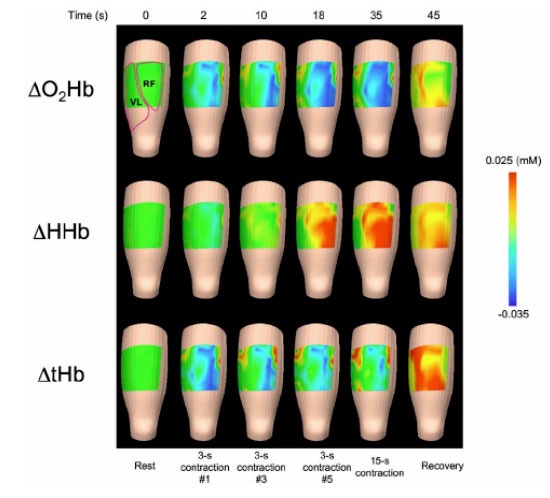
NIR DOI images from the quadriceps muscles before, during, and after intermittent isometric knee-extension exercise. The top left image indicates the approximate location of specific muscles. Contractions 1, 3, and 5 indicate images obtained during a series of 3-s duration contractions at 50% of maximum voluntary contraction (MVC), with one second rest in between. The 15-s contraction shows data at the end of a continuous 15-s MVC. The recovery image was obtained 10 s after the last contraction. These images demonstrate the spatial differences seen within muscles during exercise and recovery. O_2_Hb: oxygenated hemoglobin and myoglobin; HHb: deoxygenated hemoglobin and myoglobin; tHb: total hemoglobin and myoglobin. From Ref. [108], with permission.

Using CW imaging system similar to that shown in Figure 1, Lin et al demonstrated fast imaging of blood volume and oxygenation changes in peroneus and gastrocnemius muscles during exercise [107]. This 8-channel imager can differentiate the regions corresponding to extensors and flexors since they show different responses during exercise. Using a higher density (200-channel) CW imager which covers 45 cm × 15 cm^2^ area, Hamaoka et al recently showed DOI of quadriceps muscles before, during, and after exercise (see Figure 3) [108]. These results demonstrated the spatial differences within muscles during exercise and recovery, which would be an important tool to study muscular physiology.

Maris et al used frequency-domain NIR optical topography system to map the differences in the hemoglobin concentration in finger extensor muscle during exercise [109]. Yu et al later demonstrated a hybrid frequency-domain diffuse reflectance spectroscopy (DRS) and diffuse correlation spectroscopy (DCS) system for simultaneous monitoring of muscle hemodynamics and blood flow [110]. DRS can quantify the total hemoglobin concentration and oxygenation saturation, while DCS, an emerging extension of diffuse optical imaging techniques [111, 112], quantifies the relative blood flow in deep tissues. Together, this hybrid technique provides a method for evaluation of muscle microcirculation and metabolism in vivo.

Time-domain methods have also been extended into muscle imaging. Hillman et al have used the 32-channel time-domain DOT system to measure the absorption changes of human forearm in response to finger flexion exercise [113]. Zhao et al also developed a time-resolved (TR) DOT system and demonstrated the capability of imaging the forearm during hand-gripping test [114]. The group at Milan has developed a compact and fast multi-channel TR DOI system to image the calf muscle oxygenation and hemoglobin content during dynamic plantar flexion exercise [115]. These results strengthen the role of DOI as a powerful tool for investigating the spatial and temporal features of muscle physiology. These above results clearly demonstrated that NIR diffuse optical imaging has been widely used for imaging muscle functions and diseases. Although it is difficult to decouple the relative contributions from hemoglobin and myoglobin in the muscle [116, 117], DOI will continue to play an important role in imaging muscle functions for athletic training [118, 119] and disease diagnostics [120].

### 2.2 Optical Coherence Tomography (OCT)

#### Principle and Instrumentation of OCT

OCT is an emerging medical imaging technology which enables cross-sectional imaging of tissue microstructure in situ and in real time [121]. OCT can achieve 1–10 μm resolutions and 1–2 mm penetration depths, approaching those of standard excisional biopsy and histopathology, but without the need to remove and process tissue specimens [122]. OCT is analogous to ultrasound imaging, except that imaging is performed by measuring the echo time delay and intensity of back-reflected or backscattered light rather than sound. OCT imaging can be performed fiber-optically using delivery devices such as handheld probes, endoscopes, catheters, laparoscopes, and needles which enable non-invasive or minimally invasive internal body imaging [123].

OCT measurements are performed using a Michelson interferometer with a low coherence length light source. One arm of the interferometer illuminates the light on the tissue and collects the backscattered light (typically referred to as “sample arm”). Another arm of the interferometer has a reference path delay which is scanned as a function of time (typically referred to as “reference arm”). Optical interference between the light from the sample and reference arms occurs only when the optical delays match to within the coherence length of the light source. This technique is generally referred to as “time-domain OCT”. Alternatively, OCT interference signals can be detected in frequency or Fourier domain. In Fourier-domain OCT, the reference mirror position is fixed, and echoes of light are obtained by Fourier transforming the interference spectrum. These techniques are somewhat analogous to Fourier transform spectroscopy and have a significant sensitivity and speed advantage compared to time-domain OCT because they measure the optical echo signals from different depths along the entire axial scan simultaneously rather than sequentially. Fourier-domain detection enables 10–100 folds improvement in detection sensitivity and speed over the time-domain configuration [124–126]. These advances not only greatly improve the performance of OCT, but enables three-dimensional OCT (3D-OCT) imaging in vivo. Fourier-domain OCT can be performed using two complementary techniques, known as spectral / Fourier domain OCT and swept source / Fourier domain OCT (SS-OCT, also known as Optical Frequency Domain Imaging, OFDI). Three-dimensional imaging of biological tissue in vivo enabled by Fourier-domain OCT promises to have a powerful impact in disease diagnosis [127, 128].

To image internal organs, miniaturized catheter/endoscope imaging devices have been developed for intraluminal and intravascular imaging [129, 130]. Other imaging devices such as laparoscopes [131, 132] and needle imaging device have been developed to enable solid organ imaging [133, 134]. Nowadays, various OCT imaging probes have been developed for different clinical applications. Development of such devices facilitates the translation of OCT to clinical applications and allows clinicians to use the enhanced imaging capabilities of this technique to benefit the patients.

#### Clinical Applications of OCT

OCT was first demonstrated in 1991 [121]. Since then, OCT has rapidly developed as a non-invasive biomedical imaging modality that enables cross-sectional visualization of tissue microstructures in vivo [135–138]. The resolution of OCT is one to two orders of magnitude higher than conventional ultrasound, approaching that of histopathology, thereby allowing architectural morphology to be visualized in situ and in real time. OCT enables imaging of structures in which biopsy would be hazardous or impossible, and promise to reduce the sampling errors associated with excisional biopsy. OCT has been increasingly translated from bench to various clinical applications including ophthalmology [139–145], cardiology [146–149], gastroenterology [150–156], dermatology [157], dentistry [158, 159], urology [160–163], gynecology [164–166], among others. The most developed clinical OCT applications are those focusing on ophthalmic, cardiovascular, and oncologic imaging.

##### Ophthalmic Imaging

OCT was first applied for imaging in the eye [167, 168]. To date, OCT has made the largest and most significant clinical impact in ophthalmology. OCT provides cross-sectional views of eye with high resolution, thereby allowing detailed structures to be discerned. The non-contact and non-invasive nature of OCT enables a new way to image the structures in the anterior eye and retina, and reveal the information not available through standard ophthalmoscopes [169, 170]. Ophthalmic OCT was first commercialized by Carl Zeiss Meditec, Inc., and is now considered superior to the current standard of care for the evaluation of many retinal diseases. Over 6000 units of Status OCT™ system has been sold to date, and it is estimated that more than 37,000 OCT scans are performed daily in the U.S. With the development of high-speed OCT using spectral/Fourier domain methods, several companies have introduced new OCT instruments into the ophthalmic market in the past few years. The increased availability of high performance OCT will enable a wide range of clinical studies.

The high axial resolution of OCT makes it an ideal imaging modality for the evaluation of fine features such as intra-retinal layers and the vitreal-retinal interface. OCT has been demonstrated for the detection and monitoring of a variety of macular diseases [171], including macular edema [172–175], macular holes [142, 143, 176], central serous chorioretinopathy [177], age-related macular degeneration and choroidal neovascularization [178]. OCT can also image and quantify the retinal nerve fiber layer thickness, which is a predictor for early glaucoma. Quantitative nerve fiber layer thickness has been measured with OCT, and correlated with the optic nerve structure and function [179–184].

The increasing impact in clinical medicine promotes the rapid development in OCT imaging technologies, which dramatically enhance the imaging performance and clinical utilities of OCT. A comprehensive review of the state-of-the-art ophthalmic OCT has been provided elsewhere [185]. Here we provide a concise overview of these technology advances and their translation into ophthalmic applications.

**Figure 4:**
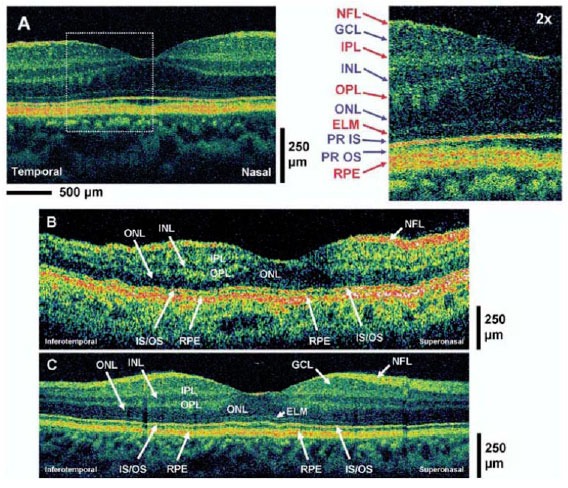
***(****A) Improved interpretation of intraretinal layers using ultrahigh-resolution OCT (UHR OCT). (B) Standard resolution OCT (10 μm axial resolution performed with a commercial OCT system) versus (C) ultrahigh resolution OCT (UHR OCT) with 3 μm axial resolution of a patient with macular hole. All intraretinal layers, including nerve fiber layer (NFL), ganglion cell layer (GCL), inner plexiform layer (IPL), inner nuclear layer (INL), outer plexiform layer (OPL), outer nuclear layer (ONL), eternal limiting membrane (ELM), junction of the inner and outer photoreceptor segment (IS/OS), and retinal pigment epithelium (RPE), are significantly better visualized by UHR OCT. From Ref. [191], with permission.*

One of the key parameters in OCT imaging is axial resolution. This is of particular interests in retinal imaging owing to the layered structures of the retina. Enhanced axial resolution enables better visualization of the intraretinal structural details and more accurate diagnosis of diseases. The axial resolution of OCT is inverse proportional to the bandwidth of the low-coherence light source. Therefore, increasing the bandwidth of the light source enables the improvement in axial resolution [186, 187]. Ultrahigh resolution (UHR) OCT achieves superior axial image resolutions of 2–3 μm as compared to ∼ 10 μm in standard OCT systems using superluminescent diode (SLD), thereby enabling the visualization of intraretinal structures [188]. UHR OCT is a key technology advance towards achieving non-invasive optical biopsy of the human retina. UHR OCT technology has been investigated in clinical settings to assess its clinical utility. Cross-sectional studies in ∼1,000 eyes with different pathologies demonstrated unprecedented visualization of all major intraretinal layers especially the photoreceptor layer [145, 189–193]. All intraretinal layers, especially the inner and outer photoreceptor segment, are significantly better visualized by UHR OCT (see Figure 4). These studies demonstrated visualization of photoreceptor layer impairment in macular pathologies such as macular holes, central serous chorioretinopathy, age related macular degeneration, foveomacular dystrophies, Stargardt’s dystrophy and retinitis pigmentosa [185]. Therefore, UHR OCT holds strong potential to enhance early diagnosis by detecting subtle morphological changes in a wide range of retinal diseases.

Transverse resolution is also an important imaging parameter. The transverse resolution is determined by the numerical aperture (NA) of the focusing lens. For ophthalmic retinal imaging, the cornea and lens act as the “imaging objective”. In practice, ocular aberrations limit the minimum focused spot size on the retina. Adaptive optics (AO) is a promising approach to correct ocular aberrations in order to decrease the spot size on the retina to improve the OCT transverse resolution [194, 195]. Combining AO with UHR OCT provides isotropic high resolution in the 3D dataset, thereby enabling cellular resolution retinal imaging [196–199]. Ultrahigh transverse resolution imaging can also be achieved by using high NA objectives (also called optical coherence microscopy - OCM) [200]. Parallel detection using full-field OCM has been demonstrated on cellular-level imaging of cornea and retina tissues [201, 202]. This technology has shown strong promises in clinical translation for in vivo ophthalmic imaging.

**Figure 5:**
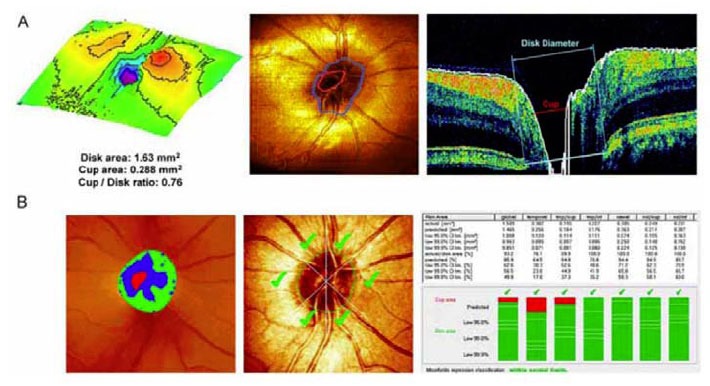
Topography using three-dimensional, ultrahigh-resolution OCT. Quantitative topography using ultrahigh-resolution 3D-OCT (A) of a normal human optic disk, as compared to those performed by Heidelberg retinal tomography (Heidelberg Engineering, Germany) (B). From Ref. [213], with permission.

Imaging speed is another critical parameter in clinical OCT imaging. High speed imaging not only reduces the motion artifacts, but enables comprehensive visualization of the three-dimensional structures. Fourier-domain OCT is a key enabling technology which dramatically increase the OCT imaging speed for three-dimensional (3D) imaging in vivo. Spectral OCT, commonly operates at 800 nm, has been rapidly developed and translated into retinal imaging. The first demonstration of retinal imaging using spectral OCT was performed in 2002 [203], and high-speed video-rate imaging was achieved 2003 [204, 205]. Three-dimensional ultrahigh-resolution ophthalmic imaging in vivo has been demonstrated on numerous clinical studies [206–210]. Three-dimensional OCT provides quantitative measurement of intraretinal layers for early diagnosis of diseases such as glaucoma or diabetic retinopathy, and enables assessment of disease progression or response to therapy. Figure 5 shows an example of topographic information of optical disk similar to that obtained by scanning laser tomography system. In addition, spectral OCT systems working at 1000 nm range promise to improve the penetration depth for better visualization of choroidal tissues [211, 212].

Other technological advancements have been applied to ophthalmic imaging as well. Doppler OCT [214–216] enables measuring of blood flow velocity in the tissue. Spectral/Fourier domain OCT with Doppler flow imaging has been demonstrate [217–219]. Three-dimensional Doppler OCT enables visualization of 3D retinal vasculature (also called optical coherence angiography [220, 221]). Another functional OCT method, polarization-sensitive (PS) OCT [222], enables detection of depth-resolved tissue birefringence and scattering properties. PS-OCT has been used for imaging the retinal nerve fiber layer (RNFL) changes in glaucoma patients [223]. This method has been applied to image both the anterior and posterior eye imaging [224–227]. Recently, significant advances has been made on detecting OCT scattering signals due to functional responses of the retina (also called optophysiology) [228]. Functional responses have been observed in vivo on human subjects [229]. This technique could detect functional impairment before morphological changes. Lastly, multi-modal technology combing OCT with other optical imaging modalities, such as scanning laser ophthalmoscopy (SLO) and fluorescence angiography promises to integrate the information from different methods and enhance the diagnostic capability [230].

##### Cardiovascular Imaging

Another major area for OCT clinical application is cardiology. The potential of OCT for cardiology imaging has been extensively investigated over the past decade [146–148, 231–239]. Compared to intravascular ultrasound (IVUS), OCT has an order of magnitude higher resolution, therefore enables visualization of fine structures in the luminal wall (such as the differentiation of intimal, medial, and adventitial layers). Several technological advances, including catheter-based imaging probes and high-speed OCT imaging, have enabled the translation of OCT cardiovascular imaging from the bench to the bedside. Development of small-diameter fiber catheters facilitates the manual feeding of the imaging catheters through the vasculature. In intravascular OCT imaging, blood will significantly attenuate the signal, which can be alleviated by saline flush or temporary vascular occlusion with balloon. To scan a long segment of an artery within a short interval of saline flush or blood occlusion, high-speed imaging is critical. Recent advancement in high-speed Fourier domain OCT provides exciting new avenue to diagnose vascular diseases and guide the intravascular interventions in real time [240].

OCT has unique ability to visualize atherosclerotic lesions in microscopic details, and in particular, to detect the vulnerable plaques which has high risks of rupture [148, 234, 236, 241, 242]. OCT can distinguish the characteristic morphology of vulnerable plaques including a thin fibrous cap and a large lipid pool, and is able to quantity the increase of macrophage activity [137, 241]. The potential of OCT imaging of vulnerable plaques was first investigated on in vitro studies and demonstrated the capability of OCT in identifying clinically relevant architectural morphology including fibrous caps, lipid-laden pools, and calcifications [243]. The specific features of different types of atherosclerotic plaques (fibrous, lipid, and calcified) can be clearly identified with histological correlation [244]. OCT images of fibrous plaques were characterized by homogeneous, signal-rich regions; fibrocalcific plaques by well-delineated, signal-poor regions with sharp borders; and lipid-rich plaques by signal-poor regions with diffuse borders. The detection sensitivity and specificity for different types of plaques are: 71–79% and 97–98% for fibrous plaques; 95–96% and 97% for fibrocalcific plaques; and 90–94% and 90–92% for lipid-rich plaques. These results represent an important step in validating intravascular OCT imaging and provide a basis for interpretation of intracoronary OCT images obtained from patients. The first in vivo coronary imaging study in humans was performed using a 1-mm-diameter (3.0 F) catheter, demonstrating the ability of intravascular OCT to visualize the coronary plaques [234], followed by subsequent study to characterize different coronary atherosclerotic plaques in vivo [149]. Intravascular OCT imaging has been investigated extensively for in vivo diagnostics of vulnerable plaques [245–248]. Identifying vulnerable plaques in situ could potentially allow cardiologists to develop therapeutic strategies, predict the vulnerability of plaques, and monitor structural changes after intervention.

OCT has also been demonstrated to be able to quantify the activated macrophage content in vivo [148]. It has been demonstrated that there was a high degree of positive correlation between OCT measurements and histological measurements of fibrous cap macrophage density. The unique capabilities of OCT for fibrous cap characterization suggest that this technology could be well suited for identifying vulnerable plaques in patients. Other quantitative analyses including the OCT signal attenuation and layer thickness changes has been undertaken for tissue characterization of OCT imaging of coronary arteries [249–251]. Recent study using both the attenuation and backscattering properties showed enhanced contrast and better tissue characterization [251]. These findings hold strong potentials for future computer-aided diagnosis of atherosclerotic plaques and better detection of thin-cap fibroatheroma (TCFA) [251].

Another potential application for intravascular OCT is to monitor therapeutic interventions, such as stent deployment [147, 252–257]. OCT provides a clear view of the stent struts and their positions relative to the wall, thereby giving surgeons a real-time assessment of stent apposition, tissue prolapse, and wall dissections. It has been demonstrated that OCT can be used to visualize stent integrity, neointimal formation, and neovascularization [238, 253]. Recent developments in drug-eluting stents promises to prevent the in-stent restenosis. Intravascular OCT imaging enables evaluation of neointimal coverage and earlier detection of excessive regrowth of the inner layer of vessel [258, 259].

In addition to high-resolution and high-speed imaging of morphology, OCT can also provide functional information for improved understanding and assessment of disease. For example, polarization-sensitive (PS) OCT has shown the potential to image intrinsic tissue birefringence changes, which can be used to differentiate fibrous and calcified plaques [260–262]. PS-OCT will significantly improve our ability to evaluate plaque stability in patients. Optical coherence elastography (OCE), another functional extension of OCT for imaging tissue biomechanical properties, has been investigated to evaluate the mechanical properties of arterial walls and plaques [263, 264]. Furthermore, a dual-modality device that combines the anatomical imaging capabilities of OCT with the functional capabilities of laser-induced fluorescence (LIF) spectroscopy has been applied for imaging normal and atherosclerotic portions of aorta wall [265]. Such dual-modal approach is desirable in cardiac functional imaging [266], where the structural heterogeneities influence the arrhythmia induction, stabilization, and termination. The comprehensive information offered by both modalities promises to characterize the patterns of functional signals and their correlation with structures in great details, thereby providing new insights into the structure-function relationships.

##### Oncology Imaging

Cancer imaging is also a promising area of OCT application. Many cancers arise from the epithelial layers, and demonstrate disruption of normal architectural morphology of tissues. The resolution and imaging field-of-view of OCT is approaching those of standard biopsy and histopathology, therefore OCT represents a potential method for “optical biopsy” of the tissue in situ, which can guide the excision biopsy to improve the sampling accuracy. OCT has shown promises in detecting structural alterations associated with malignancies including those arising in the breast [267–271], bladder [163, 272–274], brain [275–277], gastrointestinal [154, 155, 278, 279], respiratory [280] and reproductive [281] tracts, skin [282], larynx [283, 284], and oral cavity [285, 286].

**Figure 6:**
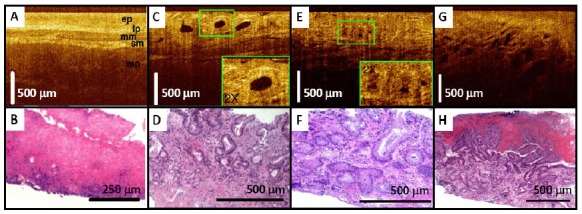
In vivo ultrahigh resolution endoscopic OCT image (top row) and the corresponding histology (bottom row). A–B: normal esophagus; C–D: Barrett’s esophagus; E–F: High-grade dysplasia; G–H: Adenocarcinoma. From Ref. [156], with permission.

Development of endoscopic OCT greatly facilitates imaging of cancers raised from internal organs in situ. OCT has been demonstrated for detection of specialized intestinal metaplasia in Barrett’s esophagus [150–152, 287] and transmural inflammation in inflammatory bowel disease (IBD) patients [288]. Recently, OCT has shown the promise for detection of high-grade dysplasia in Barrett’s esophagus. Evans et al reported 83% sensitivity and 75% specificity for detecting high-grade dysplasia and intramucosal carcinoma with blinded scoring of OCT images from 55 patients using a numeric scoring system based on the surface maturation and glandular architecture [155]. Isenberg et al reported 68% sensitivity and 82% specificity, with 78% accuracy for detection of dysplasia from 33 patients with Barrett’s esophagus [154]. Using ultrahigh-resolution (UHR) endoscopic OCT imaging with 4 μm axial resolution, Chen et al demonstrated in vivo clinical imaging in a cross-sectional study of 50 patients [156]. Real-time endoscopic OCT imaging was performed using a 1.8 mm diameter OCT catheter probe introduced into the accessory channel of a standard endoscope. Figure 6 A–B shows a representative UHR OCT image of normal esophagus which exhibits a characteristic layered architecture and its corresponding histology. C–D shows a representative UHR OCT image of Barrett’s esophagus and the associated histology. The layered architecture in normal esophagus is replaced by glandular structures. Low-backscattering Barrett’s glands are frequently observed within the mucosa, with interlaced regions of high-backscattering connective tissue corresponding to the lamina propria. E–F shows an example UHR OCT image of high-grade dysplasia and the corresponding histology. OCT images of high-grade dysplasia are characterized by irregular, distorted, and cribriform or villiform glandular architecture and are more heterogeneous than those of non-dysplastic Barrett’s epithelium. G–H shows an example UHR OCT image of adenocarcinoma and the representative histology. Irregular infiltrating glandular morphology can be visualized with OCT. Ultrahigh resolution OCT images showed good correlation of architectural morphology with histological findings. Enhanced image resolution and reduced speckle size enable ultrahigh resolution OCT to visualize tissue architectural heterogeneity more clearly than standard resolution OCT. Future clinical studies are needed to investigate the role of ultrahigh resolution OCT in detection of early neoplastic lesions.

Using Fourier-domain methods, endoscopic 3D-OCT imaging in vivo has recently been demonstrated [127, 289–291]. 3D OCT enables comprehensive assessment of early structural changes associated with diseases. With further technology refinement, higher resolution and imaging speed will be available in the near future. The unique capability of high-resolution imaging with large field of view promises to enable more complete characterization of tissue microscopic features and open new possibilities for improving identification of early neoplastic changes.

Bladder cancer is another promising candidate for endoscopic OCT imaging. Recently, a 32 patient study showed OCT has high detection accuracy for real-time imaging and staging of bladder cancer adjunct to white light cystoscopy (90% sensitivity and 89% specificity for tumor confined to the mucosa, and 100% sensitivity and 90% specificity for muscle-invasive tumors) [274]. Another clinical study with 24 patients reported an overall sensitivity of 100%, specificity of 89%, and diagnostic accuracy of 92% for OCT imaging of superficial bladder transitional-cell carcinoma (TCC) [163]. Computer-aided recognition of bladder cancer using OCT and texture analysis is under investigation to improve the clinical utility of OCT [273].

**Figure 7:**
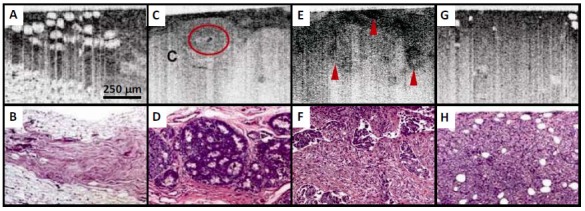
Ultrahigh resolution OCT image (top row) and the corresponding histology (Hematoxylin-eosin stain, bottom row) of human breast tissues ex vivo. (A) OCT image of normal fibroadipose tissue. (B) Histologic specimen corresponding to OCT image. (C) OCT image of DCIS lesions in lobules. (D) Histologic specimen corresponding to OCT image. (E) OCT image of infiltrating ductal carcinoma. (F) Histologic specimen corresponding to OCT image. (G) OCT image of a solid variant infiltrative lobular carcinoma. (H) Histologic specimen corresponding to OCT image. From Ref. [268], with permission.

OCT also holds promises for detection of cancers in the solid organ such as breast. In a pathological laboratory study, ultrahigh resolution OCT imaging of human breast specimens was performed in 119 freshly excised specimens from 35 women with 3.5 μm axial resolution [268]. Microstructures of normal breast parenchyma, including glands, lobules, and ducts, as well as stromal changes associated with infiltrating cancer were visible from OCT images. Furthermore, fibrocystic changes and benign fibroadenomas were identified. Imaging of ductal carcinoma in situ (DCIS) revealed microcalcifications. Figure 7 shows an example of OCT images of the human breast. Figure 7 A shows an OCT image of normal fibroadipose tissue with the corresponding histology shown in B. Fibrous stroma appears heterogeneous and highly scattering, whereas adipocytes appear low scattering, with individual, well-circumscribed scattering borders. Figure 7 C shows an OCT image of DCIS lesions in lobules. Tumor cells within lobules appear uniformly low scattering. Dilatation and architectural distortion of the lobules is visible. A microcalcification (C) within the lobules appears highly scattering with pronounced shadowing. D shows the corresponding histology. E–F shows an OCT image of infiltrating ductal carcinoma and the corresponding histology. Highly scattering regions corresponding to tongues of invasive cancer are visible. G–H shows an OCT image of a solid variant infiltrative lobular carcinoma with histology. Regions with densely infiltrating tumor cells appear low scattering and homogeneous, with isolated regions of entrapped fat. Using quantitative signal analyses including slope, standard deviation, and spatial frequency, high sensitive tissue classification has been shown with normal and tumor tissues [269, 292]. A recent 37 patient study used OCT to image surgical margins of lumpectomy specimens, yielding a sensitivity of 100% and specificity of 82% [270]. These results clearly demonstrated that OCT is a strong candidate for future clinical adoption for image-guided interventions of breast cancer including guiding breast biopsy and intra-operative margin assessment in breast-conserving surgeries. The potential of OCT in detecting cancers in other solid organs such as prostate cancer [161] and kidney cancer [293, 294] are also under investigation.

Multi-modal imaging combining OCT with other imaging techniques such as fluorescence has been actively investigated to improve the sensitivity and specificity for cancer detection. Previous studies combining OCT and laser-induced fluorescence (LIF) spectroscopy showed improved identification of tumor boundaries in the cervix [295, 296]. Tumlinson et al developed a combined OCT and LIF imaging catheter for in vivo imaging of mouse colon [297, 298]. This miniaturized 2-mm-in-diameter catheter has been applied to longitudinally monitor disease progression in the mouse colon, and is able to identify colorectal adenomas in murine models [299]. In an ex vivo study of murine GI tracts, Hariri et al showed that OCT and LIF provided complementary information for the detection of dysplasia and inflammatory bowel disease (IBD) of the intestines [300]. Pan et al showed that fluorescence-guided endoscopic OCT could enhance the efficiency and sensitivity of early bladder cancer diagnosis [301]. In a rat model study, they demonstrated that the specificity of fluorescence detection of transitional cell carcinoma was significantly enhanced by fluorescence-guided OCT (53% vs. 93%), and the sensitivity of fluorescence detection also improved by combination with OCT (79% vs. 100%) [302]. A recent clinical study involving 138 volunteers and 10 patients with lung cancer has been performed to investigate the feasibility of OCT characterization of preneoplastic changes in the bronchial epithelium identified by autofluorescence bronchoscopy [280]. The preliminary data suggested that autofluorescence bronchoscopy-guided OCT imaging of bronchial lesions is promising for in vivo imaging of preneoplastic bronchial lesions.

##### Other Applications

In addition to ophthalmology, cardiology, and cancer imaging, which are the most developed fields in OCT, novel applications are constantly being explored. OCT has shown promises in imaging cartilage diseases [303–306]. Dental OCT has also been developed extensively [159, 307–313]. Neurosurgical guidance using OCT represents another exciting new application [314]. Non-destructive evaluation of transplant kidney status using OCT [315, 316] is actively under investigation. Further research is needed to evaluate the role of OCT in a variety of clinical areas.

### 2.3 Fluorescent Techniques: Spectroscopy and Tomography

#### Principle and Instrumentation

Fluorescence techniques use fluorescence from either endogenous molecules (i.e., autofluorescence) or exogenous dyes to probe the biochemical and pathological status of the tissue [317, 318]. Typical fluorescence system illuminates the tissue with light, which excites fluorescent molecules (fluorophores) within the tissue. The emitted fluorescence light, typically at longer wavelengths than the excitation light, is collected and analyzed [319, 320]. Common endogenous fluorophores include connective tissues (collagen, elastin), cellular metabolism related coenzymes (reduced nicotinamide adenine dinucleotide (NADH), flavin adenine dinucleotide (FAD), and flavin mononucleotide (FMN)), by-products of heme biosynthesis (porphyrins), among others [321]. Several exogenous dyes also have been approved for clinical use, such as ICG [11, 40, 230, 322, 323], fluorescein [175, 324], 5-aminolevulinic acid (5-ALA) [325–327], among others. Table 1 lists the optical properties of some commonly-used fluorophores [328].

There are three types of fluorescence systems: point-probe spectroscopy, wide-field imaging, and tomography. Point-probe spectroscopy system is designed to obtain the wavelength-dependant optical properties of tissue at a single spatial location. A typical point-probe spectroscopy instrument consists of a light source, a fiber-optic delivery probe containing both the excitation and collection fibers, and a spectrometer as the detector. The excitation fiber illuminates the tissue volume and the closely spaced detection fibers collect the scattered and fluorescence light. The detected fluorescence light (after long-pass emission filter) is dispersed in wavelength by a spectrograph, and the spectrum is recorded and analyzed. The separation between the illumination and detection fibers is usually on the order of millimeters, with the total diameter of the optical probe being small enough to pass through the accessory channel of a standard endoscope. The tissue sampled in point-probe spectroscopy system is usually restricted to a small area comparable to the extent of conventional excisional biopsy.Fluorescence spectroscopy systems can be extended into wide-field imaging mode. Wide-field techniques can image over a larger surface area. The light source provides a wide-field illumination on the tissue sample and a CCD camera is used as the detector to generate high-quality images [329]. A series of emission filters can be used to acquire wavelength dependent fluorescence signals. Wide-field planar imaging typically does not contain depth-resolved information. In addition, the detected fluorescence intensities are non-linearly attenuated from different imaging depths [330–332], which could lead to a surface weighted images (in other words, signals are bias towards the surface lesions). In contrast, fluorescence tomography can reconstruct spatially-resolved fluorophore distribution, using similar approaches as diffuse optical tomography (DOT). The excitation light is illuminated from multiple source positions (through either fiber-optic coupling or scanning beam spot), and multiple detectors (such as CCD cameras) collect the fluorescence light propagating through different paths. Using image reconstruction similar to computerized tomography (CT) or DOT, the fluorophore distribution inside the tissue can be reconstructed.

#### Clinical Applications

One of the major clinical applications for fluorescence systems is early cancer detection. Several biochemical and morphologic factors may correlate with fluorescence signal changes in neoplastic lesions, including increased absorption of hemoglobin and loss of spectral contributions from submucosal connective tissues [333]. Abnormal thickening of epithelial tissue may cause attenuation of the excitation light, leading to further decreases in the fluorescence intensity. Therefore, the peak wavelength and intensity of the fluorescence spectra can be used to differentiate normal versus diseased tissues due to the changes in the concentration and distribution of metabolically related fluorophores and alterations of the tissue microstructures [317, 334, 335]. Here we focus on the applications in cancers in the gastrointestinal (GI) tracts, brain, and breast.

##### Point-Probe Fluorescence Spectroscopy

Point-probe fluorescence spectroscopy instruments can be integrated into a fiber-optic catheter device for endoscopic applications. Previous studies have shown that fluorescence spectroscopy can increase the detection rate of high-grade dysplasia (HGD) in Barrett’s esophagus [317, 334]. Using 330 nm excitation, Bourg-Heckly et al found 86% sensitivity and 95% specificity for differentiating neoplastic tissue (HGD and intramucosal carcinoma) from normal and Barrett’s mucosa in 24 patients by analyzing the fluorescence intensity ratio at 390 nm and 550 nm [334]. Panjehpour et al proposed another method called differential normalized fluorescence (DNF), where the measured fluorescence spectrum is subtracted by a baseline value (obtained by averaging the total-intensity-normalized spectra from the normal tissues) [317]. Based on the DNF intensity at 480 nm, a sensitivity of 90% and a specificity 96% for detection of HGD in non-dysplastic BE mucosa was reported from 36 patients [317]. Using the intensity ratio of green (500–549nm) to red (667–700nm) fluorescence and the intensity of blue excitation (477 nm) as two parameters for tissue classification, Mayinger et al found 97% sensitivity and 95% specificity for diagnosis of esophageal carcinoma on 9 patients [336]. Using a similar approach, they reported 84% sensitivity and 87% specificity for the diagnosis of gastric adenocarcinoma on 15 patients [337]. Fluorescence spectroscopy can also accurately identify dysplasia associated with adenomatous polyps in the colon [318, 338]. Using the probability distribution of the fluorescence intensity at 460nm and the intensity ratio of I_680nm_/I_660nm_ as the diagnostic parameters, Cothren et al reported 90% sensitivity, 95% specificity, and 90% positive predictive value (PPV) for detection of colonic dysplasia in a study with 57 patients [318]. Mayinger et al also applied light-induced autofluorescence spectroscopy for the diagnosis of colorectal cancer and adenoma [339]. In a study with 11 patients, they found 96% sensitivity and 93% specificity for rectal cancer detection, and 98% sensitivity and 89% specificity for diagnosis of dysplastic adenomas [339].

The fluorescence signal is often influenced by wavelength-dependent tissue scattering and absorption. To overcome this limitation, methods have been developed to extract the intrinsic fluorescence spectroscopy (IFS) signal by measuring the fluorescence and reflectance spectra with same illumination/detection geometry to cancel these unwanted wavelength-dependent effects [340, 341]. Using IFS, Georgakoudi et al reported 100% sensitivity and 97% specificity in differentiating HGD from low-grade dysplasia (LGD) and non-dysplastic BE on 16 patients [342].

To enhance the fluorescence detection capability for early lesions, exogenous contrast agents that can selectively accumulate in neoplastic tissues can be applied. One of the most widely used exogenous contrast agents is 5-aminolevulinic acid (5-ALA), which is converted intracellularly into protoporphyrin IX (PpIX). PpIX has greater production and retention in neoplastic cells due to the increased metabolic rate and the reduced ferrochelatase activity which converts PpIX to heme [329, 343]. As a result, the characteristic red fluorescence of PpIX is increased in neoplastic tissues [327]. Clinical studies using point fluorescence spectroscopy showed oral administration of 5-ALA can detect HGD from non-dysplastic BE [344]. Using PpIX fluorescence alone, 77% sensitivity and 71% specificity were reported in a study with 20 patients, whereas 100% sensitivity and 100% specificity can be achieved by using the fluorescence intensity ratio I_635nm_/I_480nm_[344]. To further differentiate the PpIX fluorescence from autofluorescence, Ortner et al invented time-gated fluorescence spectroscopy utilizing the long fluorescence decay time of PpIX to suppress the autofluorescence background [345]. In this approach, nanosecond excitation pulses is used to excite the tissue, and then the ratio of 20 ns delayed PpIX fluorescence intensity to the immediate autofluorescence intensity is calculated. Using this method, it was possible to differentiate LGD from non-dysplastic BE, and dysplasia can be detected at a rate of 2.8 times higher compared to white-light screening endoscopy [345].

Fluorescence spectroscopy can be combined with other spectroscopic techniques to provide complementary information about the biochemical and morphological state of tissue. Georgakoudi et al demonstrated that superior results for differentiating dysplastic from non-dysplastic epithelium can been achieved by combining fluorescence, reflectance, and light-scattering spectroscopies [342]. In such a multi-modality approach, fluorescence spectroscopy (IFS) provides the biochemical information, diffuse reflectance spectroscopy (DRS) reveals morphologic information about the bulk tissue, and light-scattering spectroscopy (LSS) indicates the nuclei size and density information. Tri-modal spectroscopy has been shown to detect HGD from LGD and non-dysplastic BE with 100% sensitivity and 100% specificity on 40 sites from 16 patients [342]. The results from tri-modal spectroscopy are better than those obtained using individual modalities alone as tri-modal spectroscopy combines the advantages of each modality. Such multi-modality methods can be extended to imaging modes to enable rapid surveillance of large tissue areas.

##### Wide-Field Fluorescence Imaging

One example of the clinically used wide-field fluorescence systems is autofluorescence endoscopy, which can be implemented by using fiber-coupled wide-field excitation and CCD cameras for detection. There are several approaches to generate fluorescence images. Wang et al used a long-pass filter (> 400 nm) to select the fluorescent light, and an intensified charge injection device (CID) camera to capture the fluorescence image [346]. To account for the non-uniform illumination/detection geometry, they applied a moving average algorithm to the acquired image. The area of dysplasia was determined by the fluorescence intensity below a certain threshold. Another approach used two filters and intensified CCD (ICCD) cameras to detect the fluorescence in the green (490–560 nm) and red (>630 nm) wavelength ranges, and the ratio between these two channels (I_red_/I_green_) was used to create pseudo-color images in real time [347, 348]. In this “laser-induced fluorescence” (LIF) system, normal mucosa usually appears cyan (blue-greenish), whereas neoplastic lesions appear red due to the higher red/green fluorescence intensity ratio. Newer generations of fluorescence endoscopy systems incorporate total autofluorescence and both green and red reflectance into the imaging algorithm. In this case, non-dysplastic mucosa appears green whereas neoplastic lesion appears blue-purple [349].

Fluorescence imaging can accurately identify dysplasia associated with adenomatous polyps in ex vivo colon specimens [346]. In an in vivo study of 30 patients, dysplasia was identified with a sensitivity of 83% [350]. Fluorescence endoscopy with LIF mode (LIFE) has been found to enhance the ability to localize small neoplastic lesions in the bronchus [351] and the GI tracts [347, 348, 352]. However, a randomized crossover study on 50 patients showed that LIFE was not superior to standard video endoscopy in detecting early neoplasia in Barrett’s esophagus (sensitivity for the diagnosis of HGD / early-stage cancer in targeted biopsy were both only 62%) [353].

As in point-probe fluorescence spectroscopy, using exogenous contrast agents such as 5-ALA promises to enhance the detection capability in wide-field fluorescence imaging. Endlicher et al found a sensitivity of >80% for dysplasia detection, while the specificity was only between 27% to 56% in a study of 47 patients [326]. Messmann et al also used 5-ALA to evaluate the detection of low and high-grade dysplasia in ulcerative colitis patients, and reported a sensitivity of 87–100%, while the specificity was only in the range of 51–62% in a study of 37 patients [325]. The high false positive rates are most likely associated with inflamed tissue or fecal materials [327, 354].

Using video autofluorescence imaging (AFI) system with both green and red reflectance, Kara et al recently demonstrated that AFI detected more dysplastic and neoplastic regions in Barrett’s esophagus than conventional endoscopy [349]. In a cohort of 60 patients with Barrett’s esophagus, 22 patients were diagnosed with high-grade intraepithelial neoplasia (HGIN), and among them, 7 patients were detected solely by AFI after high-resolution endoscopy (HRE) had not shown any suspicious lesions. However, the positive predictive value (PPV) from per lesion analysis was only 49%. Further technology development and combination of other imaging modalities will improve the specificity and PPV.

**Figure 8:**
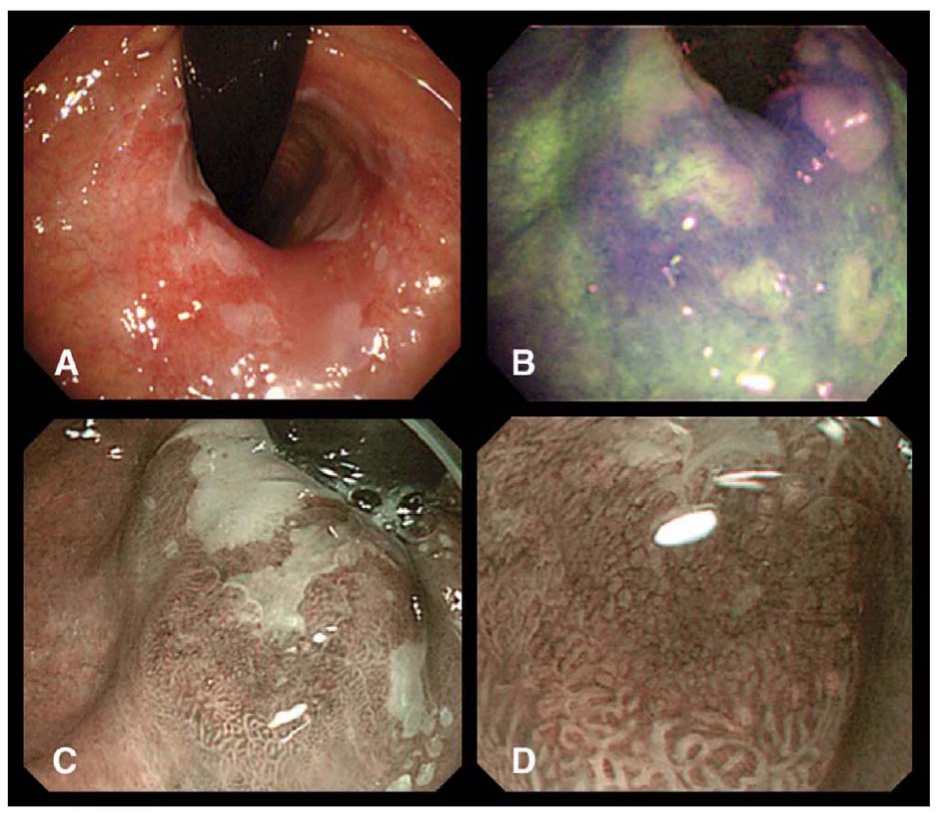
The images of a Barrett’s esophagus lesion with high-grade intraepithelial neoplasia (HGIN) detected with autofluorescence imaging (AFI) and narrow band imaging (NBI). (A) During inspection with white light, this area was not judged as suspicious. (B) The area around the small squamous island in the middle of the image showed a blue violet autofluorescence imaging color. (C) and (D) With NBI, irregular and disrupted mucosal patterns were found. The histopathology confirmed the presence of HGIN. From Ref. [359], with permission.

One of such promising modalities is narrow-band imaging (NBI). NBI utilizes a set of optical filters to allow only narrow wavelength regions of blue, green, and red light to sequentially illuminate the tissue [355–357]. Blue light penetrates only superficially, whereas red light penetrates into deeper layers. In addition, blue and green wavelengths are strongly absorbed by hemoglobin. Therefore, NBI enhances mucosal surface contrast and capillary patterns allowing detailed inspection of the mucosal and vascular patterns with high resolution and contrast without the use of exogenous dyes to improve visualization and diagnosis [358]. When combining AFI with NBI, AFI has high sensitivity in detecting dysplasia, and therefore can first scan large areas of mucosal surface to identify possible regions of neoplasia. However, AFI is often associated with high false-positive rate. NBI, on the other hand, can provide magnified inspection of mucosal patterns for detection of dysplasia. Therefore, the combination of AFI and NBI can provide complementary information for more accurate detection of early neoplasia. Kara et al performed a cross-sectional study on 20 patients with BE using endoscopic video AFI followed by NBI [359]. AFI identified all HGIN lesions (100% sensitivity), however, the false positive rate was high (19 in 47 lesions, 40%). Using NBI, the false positive rate dropped to 10% (5/47). Figure 8 shows an example of the detection of HGIN lesions using the combined modality.

**Figure 9:**
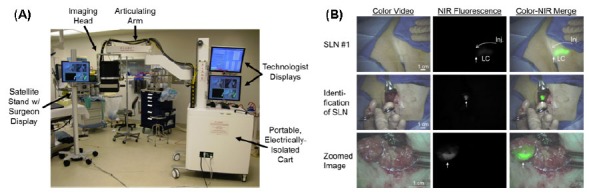
(A) Portable FLARE™ imaging system and satellite monitor stand deployed in the operating room. (B) NIR fluorescent sentinel lymph node (SLN) mapping in a woman with breast cancer. Color video images (left), 800 nm NIR fluorescence images (middle) and a pseudo-colored (lime green) merge of the two (right) after injection (Inj.) of 10 μM ICG:HSA. The single SLN identified and resected for this patient. Shown are flow through a lymphatic channel (LC) and position of the SLN (arrow; top row), identification of the SLN (arrow; middle row), and a zoomed image of the SLN (arrow) during resection (bottom row). 800 nm camera exposure time was 200 msec. From Ref. [366], with permission.

Wide-field imaging also enables image-guided resection of tumor and tumor margin assessment. Keller et al demonstrated autofluorescence and reflectance spectral imaging as a valuable tool for examining the superficial margin status of excised breast tissue specimens, with 85% sensitivity and 96% specificity [360]. 5-ALA-induced PpIX fluorescence imaging has found great success clinically in fluorescence-guided resection of malignant glioma [361, 362]. Fluorescence-guided resection improves the results of glioma surgery for gross total resection and patient survival. Several clinical trials are undergoing to further validate its clinical utility [361–365]. Another FDA approved fluorescence contrast agent is ICG. Troyan et al recently demonstrated successful 6-patient pilot human clinical trial in breast cancer sentinel lymph node mapping using an intra-operative NIR fluorescence imaging system (FLARE™) [366]. Figure 9 shows the photo of the system and an example of SLM identification using peri-tumoral injection of ICG. These results demonstrate successful clinical translation of a new NIR fluorescence imaging system for image-guided oncologic surgery. Intra-operative fluorescence imaging of ICG lymphatic draining promises to guide resection or biopsy of various forms of cancers (including breast cancer [367–371], skin cancer [372, 373], gastric cancer [374, 375], and rectal cancer [376]), monitor the perineal wound contamination in abdominoperineal resection (APR) [377], as well as to interrogate the difference between normal and abnormal lymphatic structure and function [378–382].

##### Fluorescence Tomography

**Figure 10:**
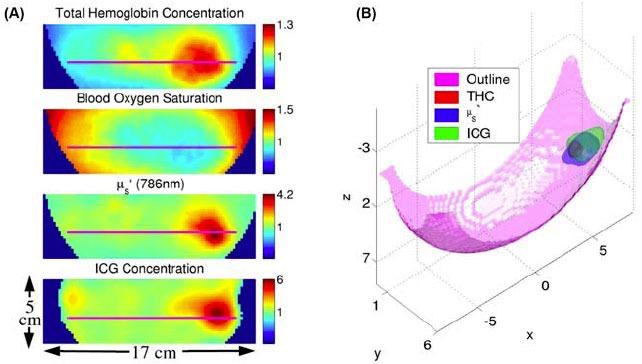
(A) Total hemoglobin concentration, blood oxygen saturation, μ_s_’(786nm) and fluorescence image slices at y = 5 cm. (B) Iso-surface plot of THC, μ_s_’ (786nm) and fluorescence at iso-values of three standard deviations above their respective means correspond to tumor location. Outline designates the border of the breast modeled as an ellipsoid using the breast photo taken with the CCD camera. From Ref. [323], with permission.

Fluorescence tomography can provide cross-sectional and volumetric views of biological tissue, therefore it is a promising tool to quantitatively characterize lesions and monitor therapy. Corlu et al reported the first human study of fluorescence tomographic imaging of breast tumor with ICG contrast enhancement [323]. Figure 10 shows the tomographic reconstruction of hemoglobin concentration, blood oxygen saturation, scattering coefficient and fluorescence signal. The reconstructed images demonstrated significant tumor contrast compared to typical endogenous optical contrast in breast, such as hemoglobin concentration and scattering coefficients obtained with traditional diffuse optical tomography (DOT). Successful fluorescence tomography in human represents a critical step towards application of molecularly-targeting probes for future clinical translation.

##### Other Techniques and Applications

Besides the spectrally-resolved steady-state fluorescence measurement techniques described above, time-resolved fluorescence measurement techniques [383, 384] are evolving and currently under investigation as a potential tool for clinical diagnosis and surgical guidance. Different from the intensity-based steady-state methods, time-resolved methods measure the fluorescence intensity decay properties (lifetime) to provide additional information of tissue. There are several advantages of using time-resolved fluorescence systems to investigate biological tissues [385]:

1. Biomolecules with overlapping fluorescence emission spectra but with different fluorescence decay times can be discriminated.
2. The measurements are sensitive to various parameters of the biological microenvironment, including pH, ion concentration and binding, enzymatic activity, and temperature, thus allowing these variables to be analyzed.
3. Time-resolved measurements are more robust to changes in fluorescence excitation-collection geometry; presence of endogenous absorbers (e.g., hemoglobin); photobleaching; and changes in fluorophore concentration and location depth, light scattering, and excitation intensity.

Time-resolved fluorescence techniques have been applied to detect atherosclerotic plaques and shown great promise in providing diagnostic information for high-risk atherosclerotic plaques [385]. In addition, applications such as tumor detection [386, 387] and image-guided tumor surgery [388] are actively pursued. With further technical development and pilot studies, this method holds strong promise on clinical translation.

### 2.4 Optical Molecular Imaging

#### Probe and Instrumentation

Nonspecific contrast agents (such as ALA and ICG) passively accumulate in diseased tissues without a specific molecular targeting moiety, therefore are subject to low sensitivity and specificity. Recently, there have been great advances in targeted imaging of basic molecular processes such as gene expression, enzyme activity, and disease-specific molecular interactions in vivo [389–391]. Molecular imaging promises early detection and in situ characterization of diseases with high sensitivity and specificity [392–395]. Optical imaging in conjunction with near-infrared fluorescent imaging probes has been developed to improve early disease detection [396, 397]. Generally, there are two major strategies for molecular-targeting contrast agents [398], including active targeting (using targeted molecular probes with high affinity for specific disease-related targets) and selective activation of the image probe at target tissues.

##### Affinity-Based Molecular Probes

Affinity-based molecular probes utilize the molecular selectivity of diseased cells to differentiate normal from abnormal tissues. Compared to normal cells, diseased cells tend to over-express certain specific molecular biomarkers, therefore providing a means for imaging contrast. A variety of targeting moieties can be used to deliver the reporter dyes to the diseased tissue. Representative strategies for reporter dye-labeled molecular targeting contrast agents are listed in Table 2. Common approaches include: monoclonal antibodies [399, 400], protein ligands [401–404], small peptides [405–407], and non-peptide ligands [408–410].

##### Activatable Molecular Probes

**Figure 11:**
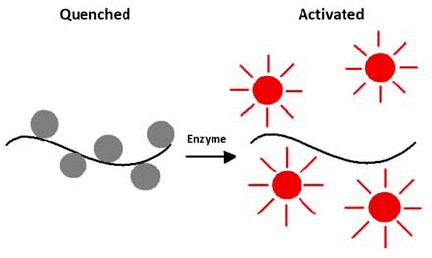
Principle of enzyme activated molecular probe.

Activatable probes are initially administered in a quenched (non-fluorescent) state, and the fluorescence signals increase when the probes are activated by specific biomolecules or environment in the disease tissue. Figure 11 shows an example of enzyme activatable imaging probe. Originally, the fluorophores are stacked together on a polymeric support, which leads to the quenching of fluorescence signals through Forster resonance energy transfer (FRET). The peptide linker is recognized by a specific proteolytic enzyme. Upon linker cleavage by the enzyme, the fluorophores are detached, which leads to dequenching and increases the fluorescence signal. Diseases tissues tend to have higher level of certain enzyme classes, therefore activatable probes provide an imaging contrast to differentiate normal from diseased tissues. Examples of activation mechanisms include [432]: enzymes, nucleic acids, ions and reactive oxygen species (ROS). Table 3 listed some representative activatable molecular imaging probes.

##### Optical Molecular Imaging Systems

Optical molecular imaging systems are essentially similar to those used in fluorescence imaging. Fluorescence imaging can be performed with different resolutions and penetration depths ranging from microscopy to tomography. Fluorescence reflectance imaging (FRI) is commonly used for two-dimensional mapping of superficial fluorophore distributions. Fluorescence molecular tomography (FMT) [332] enables three-dimensional quantification of fluorescence signals inside scattering tissues. Using near-infrared fluorophores, deeper penetration can be achieved. In addition, tissue auto-fluorescence is reduced at longer wavelength, thereby improving target to background ratio.

Miniaturized endoscope devices can be developed for molecular imaging inside luminal structures in vivo. For example, Funovics et al developed a miniaturized 2.7 F (0.8 mm in diameter) fiber-optic sensor for laparoscopic imaging of enzyme activity and gene expression in vivo [445]. This device includes a dichroic mirror, a bandpass filter, and two independent cameras permitting simultaneous recording of white-light and fluorescent images. Zhu et al also demonstrated a one-dimensional near-infrared fluorescence imaging catheter for the detection of atherosclerotic plaque in human carotid plaque specimens *in vitro*[446]. The endoscopic devices will enable the intraluminal molecular imaging of GI tracts and vessels for early diseases identification.

#### Applications and Clinical Translation

##### Oncology

**Figure 12:**
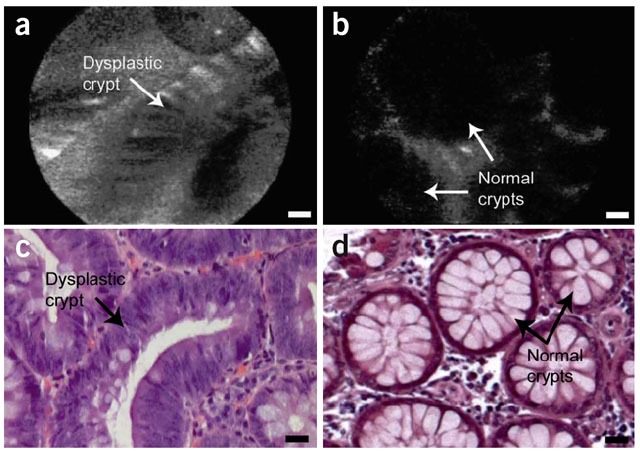
In vivo confocal fluorescence images of peptide binding. (a) Binding to dysplastic colon polyp. (b) Binding to adjacent normal mucosa. (c,d) Histology of dysplastic colon polyp (c) and normal mucosa (d) stained with H&E. Scale bars, 20 μm. From Ref. [449], with permission.

Numerous biomarkers for cancers have been identified to facilitate early detection [447]. Antibodies to these markers have high specificity, but their in vivo use has been limited by immunogenicity [448]. In contrast, peptides are typically less immunogenic, non-toxic, and relatively easier for mass production. Kelly et al developed fluorescent affinity ligands derived from a phage library specific for colon cancer, and demonstrated a 7-fold higher contrast than a control in orthotopic colonic tumors (HT29) using a two-channel miniaturized near-infrared fluorescent endoscopy [396]. Hsiung et al screened phage display peptide libraries against fresh human colonic adenomas for high-affinity ligands with preferential binding to premalignant tissue [449]. Furthermore, they conjugated the peptide with fluorescein and topically applied to human patients undergoing colonoscopy. In vivo fluorescence confocal microendoscopy images showed stronger binding of fluorescent affinity ligands to dysplastic tissues than normal (see Figure 12), with an overall sensitivity of 81% and specificity of 82%. Phage display based molecular imaging and targeted therapy represents a promising diagnostic and therapeutic approach for early detection of colorectal cancer [450] and other cancers, such as prostate cancer [451], cholangiocarcinoma [452], hepatocarcinoma [453], and melanoma [454], among others.

In addition, proteolytic enzymes have been shown to play an essential roll in tumor growth, including high cell turnover, invasion, and angiogenesis [455]. Cathepsin B in particular has been shown to be up-regulated in areas of inflammation, necrosis, angiogenesis [455], focal invasion of colorectal carcinomas [456], and dysplastic adenomas [457, 458]. Marten et al applied the fluorescent enzyme-cleavable and activatable cathepsin B sensing probe, which is non-fluorescent at injection and locally activated after target interaction, to the APC^min^ mouse model [397]. Ex vivo fluorescent imaging showed increased detection sensitivity and specificity, and the smallest lesion detected measured about 50 μm [397]. In vivo imaging of enzyme activity for colorectal cancer detection can be achieved using catheter-based microendoscope [445, 459]. Protease activity concentration (PAC), quantified by fluorescence molecular tomography (FMT), has been demonstrated to be a unique in vivo diagnostic parameter for tumor detection and chemotherapy monitoring for brain glioma [460]. Another family of proteases, the matrix metalloproteinase (MMP), also shows higher expression in cancers than normal tissues. Studies have indicated that MMP-2 degrades the extracellular matrix and is involved in tumor infiltration and angiogenesis [461, 462]. Clinical studies also show a correlation between the level of MMP-2 expression and poor disease outcome [463]. Therefore, in vivo imaging and quantification of MMP-2 expression would be important in characterization of tumors. Bremer et al developed an activatable probe which can sense the MMP activity in vivo using near-infrared optical imaging [435, 436].

Mucins (glycoproteins that cover the surfaces of epithelial cells and aid the epithelia in homeostatic and metabolic functions) represent another promising target for molecular imaging. Colorectal tissues are abundantly supplied with mucins throughout the mucosa; however, the adenoma to carcinoma transformation of cancerous cells alters O-glycans mucinous expression [464]. During carcinoma transformation of cells, O-glycans mucinous expression is altered in tumor tissues [465]. An earlier study found out that “an exposed carbohydrate structure that is not normally present in human tissues is expressed in the mucin produced by malignant colonic epithelium” [466]. Another study also reported the levels of mature Muc1 mucins were significantly higher in carcinoma tissues than those in normal mucosa (p<0.001) [467]. A more recent study showed 100% Muc1 expression in colonic adenocarcinomas and 76% expression in adenomas, relative to 29% Muc1 expression by mucosa within 2 cm of the cancer margin, and 0% expression by normal mucosa > 2 cm from the cancer margin [468]. Previous studies demonstrated that α-L-fucose binding lectin Ulex europaeus agglutinin-1 (UEA-1) showed positive binding in human colorectal specimens of adenocarcinomas, adenomas, and polyposis coli, but not in the normal epithelium [469, 470]. In addition, increased UEA-1 reactivity in polyposis patients with a familial history of large bowel carcinoma has been reported [471]. Furthermore, it was reported that there was an 83% positive rate of UEA-1 binding on apical surfaces of human carcinoma cells, compared with a 0% positive rate of UEA-1 binding on non-neoplastic mucosa adjacent to the carcinoma [472].

**Figure 13:**
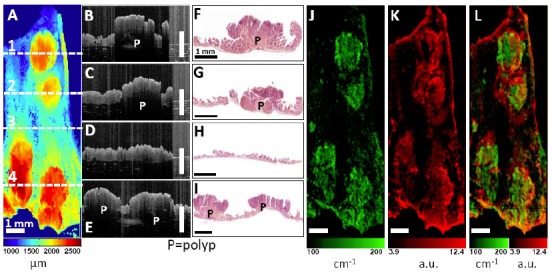
Co-registered OCT/FMI of intestinal polyps ex vivo. (A) OCT en face surface profile image. (B–E) Cross-sectional OCT images corresponding to lines 1–4 in (A) and corresponding histology (F–I). (J) Tissue scattering coefficient (μ_s_) image. Polyps show higher scattering coefficients. (K) Fluorescence image using the contrast agents targeting to α-L-fucose over-expressed in the mucin of polyp regions. (L) Fused scattering coefficient and fluorescence image. Bar = 1 mm. From Ref. [474], with permission.

Roney et al developed UEA-1 conjugated polymerized liposomes for fluorescence molecular imaging (FMI) [473]. Figure 13 shows the results of the OCT/FMI imaging of APC^min^ mouse intestine ex vivo [474]. Polyps are visible in OCT images (Figures 13A–E) and histology (F–I). Fluorescence intensities (13K) are higher around the polypoid areas. This indicates the preferential accumulation and targeted binding of UEA-1 conjugated contrast agents to the polyps (13L).

Hypermetabolism is another hallmark of cancer and has been utilized clinically for radiolabeled fluorodeoxy glucose (^18^FDG) based positron emission tomography (PET) imaging for cancer detection [475]. The analogous fluorescent deoxy glucose (2-NBDG) has shown enhanced fluorescence in neoplastic tissues compared to normal tissues [408]. NIR fluorescence labeled glucose analogs also showed in vivo tumor-to-normal contrast enhancement [409, 410, 428–430, 476] and efficient photodynamic therapy [431]. Although the mechanism of cellular uptake is still under debate [477], metabolism-based molecular probe holds the promise of addressing the universal nature of cancer cells (not limited by specific cancer types) and could be an optical surrogate of FDG [394].

The expression of folate receptor-α (FR-α) is increased in 90–95% of epithelial ovarian cancers (EOC) [478, 479], and therefore could be a promising target for molecular imaging. Recently, van Dam et al performed the first in-human intra-operative fluorescence-guided ovarian cancer surgery using tumor-specific FR-α-targeted fluorescence contrast agent [480]. This work shows the potential application of molecular imaging in patients with ovarian cancer for improved intra-operative surgical guidance.

##### Skeletal Disease

Optical molecular imaging is also able to image osteoblastic activity. The development and integrity of the skeleton requires hydroxyapatite (HA) deposition by osteoblasts. Zaheer et al developed a NIR fluorescent bisphosphonate derivative which specifically binds to HA, thereby revealing osteoblastic activity in living animals [481]. Recently, Kovar et al synthesized a NIR fluorescent tetracycline derivative which binds specifically to differentiated mineralized osteoblasts [482]. Those agents hold promises in imaging bone development and mineralization, osteoblastic metastasis, and bone remodeling process.

Using FMT, Zilberman et al imaged fracture repair on murine models with implantation of mesenchymal stem cells overexpressing the osteogenic gene BMP2 [483]. Real-time imaging and quantification of bone formation and remodeling was performed using bisphosphonate imaging agent (OsteoSense™). Higher fluorescence signals was found at implantation sites, indicating fluorescence molecular imaging has the potential for quantitative evaluation of bone regeneration and tissue engineering.

Optical molecular imaging is also able to image and characterize arthritis, including rheumatoid arthritis (RA) and osteoarthritis (OA). Chen et al developed a NIR fluorophore-conjugated folate as probe for RA imaging [484]. A fluorescence signal intensity ratio of 2.3 between arthritic and normal joint was detected 12 and 24 hours after folate injection, which shows the potential for early diagnosis of RA. In addition, proteinase activities are altered during arthritis. Wunder et al used an activatable probe to detect proteinase activity in joints [485]. Similar approach also performed by Lai et al to measure the proteinase activity in OA instead of RA [486]. Together, these studies demonstrated the potential of optical molecular imaging as a means for mechanistic study and clinical applications for orthopedic research [487].

##### Cardiovascular Disease

Optical molecular imaging can visualize molecular targets rather than anatomic structures therefore helps to elucidate the underpinning molecular and cellular mechanisms associated with cardiovascular diseases in vivo. NIR fluorescence molecular imaging has been applied to image atherosclerosis in vivo [488]. Chen et al. used FMT to image protease activity (ProSense™) in atheromata [489]. Non-invasive FMT detected the fluorescence signal in the atherosclerotic aorta in vivo and correlated well with ex vivo FRI images. This study demonstrated the feasibility of FMT to visualize augmented plaque protease activity. Deguchi et al investigated MMP-2 and MMP-9 activity in atherosclerosis using FMT [490]. Augmented NIR fluorescence (NIRF) signals were detected and co-localized with macrophage accumulation. As macrophages contribute pivotally to cardiovascular diseases, in vivo imaging of macrophages and protease activity would provide an important means to understanding the pathphysiology, evaluating the effects of interventions, and ultimately aiding clinical care [491].

**Figure 14:**
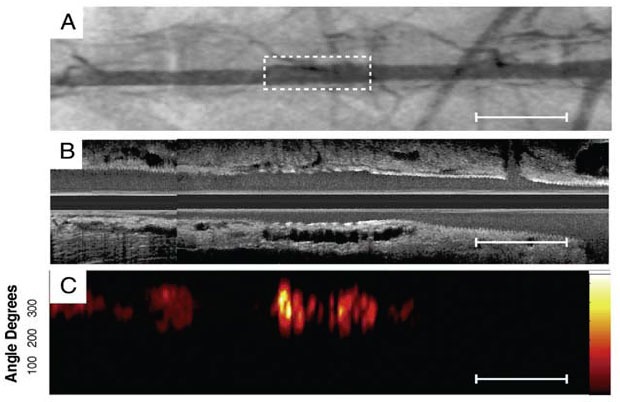
Representative multimodality NIRF molecular and IVUS anatomical imaging of arterial inflammation at day 7 following coronary stent implantation. (A) Angiogram of an implanted bare-metal stent in the abdominal aorta. Dotted rectangle denotes stent position. (B, C) Longitudinal IVUS and NIRF catheter pullbacks demonstrate NIRF signal within the stent. NIRF signal collection was performed through blood without flushing, in 3.5-mm diameter vessels. From Ref. [493].

To facilitate the clinical translation of molecular cardiovascular imaging, Jaffer et al developed an intravascular NIRF molecular sensing catheter based on the OCT imaging catheter [492]. This one-dimensional intravascular fluorescence catheter can detect cysteine protease activity using ProSense™ in real time. This device could aid in the detection of inflammation and high-risk plaques in small arteries. To provide a two-dimensional (2D) imaging, Jaffer et al also developed a 2D intravascular NIRF imaging catheter using rational and pullback design [493]. In atherosclerosis, 2D NIRF imaging provided insight into the spatial distribution of plaque protease activity. In stent-implanted vessels, 2D NIRF imaging indicated an edge-based pattern of stent induced arterial inflammation. Figure 14 shows in vivo multimodality x-ray angiography, IVUS, and intravascular NIRF imaging [493]. In stent-injured vessels, increased in vivo NIRF signal localized at the edges of the implanted stents, and particularly at the leading or distal stent edge (Fig. 14C), suggesting that stent-based injury occurred at sharp transition points. The continued development of molecular-sensing imaging agents and minimally-invasive intravascular fluorescence imaging device promise to provide high-resolution in vivo spatial mapping of inflammation-regulated protease activity in vivo.

## 3. Conclusion

This paper provides an overview of several emerging optical technologies, including their principles and translation to various biomedical applications. The continuous technology development in advanced light sources, miniaturized imaging devices, and detection methods, will motivate future clinical investigations to determine optics method’s utility in medicine. The future technology development will be strengthened by the translational research from the bench to the bedside, and the clinical feedback will shape the new technology advancement. In addition, a close academia-industry synergy will facilitate the technology transfer and bring the new optical technologies to the hands of the clinicians. With such inter-disciplinary collaboration, optical methods are promised to significantly impact the future practice in clinical medicine.

## Figures and Tables

**Table 1.** Optical Properties of Representative Fluorophores.

Fluorophore	Excitation (nm)	Emission (nm)
NADH	290–370	340–460
FAD	430–460	460–520
Collagen & Elastin	300–370	400, 440
Protoporphyrin IX	410	635
Fluorescein	494	521
ICG	805	835

**Table 2.** Representative Affinity-Based Molecular Probes.

Targeting Moiety	Biomarker	Reference
Monoclonal Antibodies (mAb)	VEGFR, VEGFR2	[399, 400]
Protein Ligands	VEGFR	[401, 402]
	EGFR	[403, 404]
Small Peptides	Somatostatin	[405–407, 411, 412]
	Integrin	[413–420]
	Bombesin	[421–423]
Non-Peptide Ligands	Folate receptor	[424–426]
	Glucose transporter	[408–410, 427–431]

VEGFR: vascular endothelial growth factor receptor; EGFR: epidermal growth factor receptor

**Table 3.** Representative Activatable Molecular Probes.

Activation Mechanism	Biomarker	Reference
Enzymes	Cathepsin	[433, 434]
	MMP	[435–437]
Nucleic Acids	RNA	[438, 439]
Ions	Calcium	[440]
	pH	[441, 442]
ROS	H_2_O_2_	[443, 444]

MMP: metalloproteinase; ROS: reactive oxygen species
